# Manifesto by the editors of Brazilian scientific journals in the field of human aging in support of open access to research and publications

**DOI:** 10.1590/1980-5764-DN-2026-E001

**Published:** 2026-09-07

**Authors:** Patrick Alexander Wachholz, Adriano Pasqualotti, Alfredo Cataldo, Anelise Crippa, Beltrina Côrte, Carla Helena Augustin Schwanke, Einstein Francisco de Camargos, Graciela de Brum Palmeiras, Johannes Doll, Kenio Costa de Lima, Lilian Cristine Hubner, Maria Elisa Gonzalez Manso, Régis Gemerasca Mestriner, Renato Gorga Bandeira de Melo, Ricardo Nitrini, Roberto Alves Lourenço, Sonia Maria Dozzi Brucki, Vanessa Sgnaolin, Renata Eloah de Lucena Ferretti-Rebustini

**Affiliations:** 1 Revista Brasileira de Ciências do Envelhecimento Humano, Passo Fundo RS, Brazil.; 2 Pan-American Journal of Aging Research, Porto Alegre RS, Brazil.; 3 Kairós Gerontologia, São Paulo SP, Brazil.; 4 Geriatrics, Gerontology and Aging, Rio de Janeiro RJ, Brazil.; 5 Estudos Interdisciplinares Sobre o Envelhecimento, Porto Alegre RS, Brazil.; 6 Revista Brasileira de Geriatria e Gerontologia, Rio de Janeiro RJ, Brazil.; 7 Dementia & Neuropsychologia, São Paulo SP, Brazil.

The academic community has been dedicated to understanding and incorporating the *modus operandi* of Open Science (OS) into the practices of knowledge production and dissemination. The United Nations Educational, Scientific and Cultural Organization (UNESCO) defines OS as a movement aimed at making scientific knowledge openly available, accessible, and reusable, grounded in key pillars that include open access to publications, data and code sharing, shared infrastructure, societal engagement, and scientific integrity.^1^

The rapid advancement of science requires that OS practices be adopted and consolidated throughout the entire research cycle. Therefore, it is essential that scientific journals commit to implementing OS best practices. This document represents a manifesto by the editors of Brazilian journals dedicated to publishing research on aging, advocating for the adoption and implementation of OS principles in their editorial processes.

## A CALL FOR A PARADIGM SHIFT

The accelerated aging of the global population presents multiple challenges across all sectors of society, including health care systems and academic and research institutions. Addressing the challenges inherent in the aging process in Brazil and worldwide requires a reorganization of research processes and practices to meet the specific needs of this population. In this context, OS emerges as essential because its principles and practices support the production of high-quality, evidence-based knowledge that is accessible, shareable, verifiable, ethical, responsible, and socially relevant.

## OPEN SCIENCE IN RESEARCH ON HUMAN AGING

Aging, as a heterogeneous and multidimensional phenomenon, demands transdisciplinary collaboration in the production and dissemination of scientific knowledge. Research approaches that fail to incorporate diverse perspectives limit comprehensive understanding. In this regard, OS principles can foster a culture of collaboration and sharing, promoting knowledge generation at the forefront of science through the integration of researchers and multiple sectors of society.

The adoption of the *modus operandi* of OS aims to ensure free and unrestricted access to research findings for both researchers and society at large.^2^ It enhances transparency in research methods and in the content of the data collected, strengthens reproducibility, and promotes the use of advanced methodologies through the sharing of open research outputs. Furthermore, it supports responsible research assessment and encourages the inclusion of diverse societal perspectives, giving voice to older adults and their communities, thereby strengthening public trust in science.

## THE ROLE OF SCIENTIFIC JOURNALS

Scientific journals play a strategic role as primary vehicles for disseminating academic knowledge, serving as formal channels that validate the quality, originality, and relevance of research prior to publication. Through the editorial process, journals receive, evaluate, and disseminate research findings based on rigorous methodologies reviewed by experts in the field. Editors are thus stewards of high-quality scientific communication and key agents of change.

By adopting editorial practices aligned with OS principles, journals can strengthen the culture of science popularization and ensure free and unrestricted access to shareable and verifiable results, data, and code. The adoption of responsible research assessment practices also reinforces the principles of the San Francisco Declaration on Research Assessment (DORA).^3^ The expected outcome is an expansion of the impact of scientific production and the democratization of knowledge, enabling new insights into aging to reach society at large.

## SOCIAL COMMITMENT

Access to information is a fundamental right established in the Universal Declaration of Human Rights.^4^ This prerogative should extend to scientific research outcomes, ensuring that relevant discoveries are shared with society to promote collective development. Regarding human aging research, these precepts are fundamental, as they are inseparable from the principles of equity and social justice. In this scenario, OS plays a decisive role in mitigating disparities in both the production and access to scientific knowledge, particularly in countries such as Brazil. By facilitating the inclusion of less-resourced nations in global research infrastructures, OS consolidates the democratization of knowledge as an ethical imperative for researchers. Scientific journals therefore bear the responsibility to promote and safeguard these practices.

## IN THE NAME OF A NEW EDITORIAL POLICY

Editors of scientific journals are encouraged to adopt OS principles in their editorial processes and practices. They should take a leading role in integrating OS into editorial workflows, in the name of a new editorial culture that is more equitable, transparent, and accountable. If OS catalyzes innovation, journal editors must act as agents of change. Research on aging, particularly that addressing transdisciplinary, sustainable, dignified, and high-quality care, should be developed and disseminated openly to advance society.

## PROMOTING EDITORIAL PROCESSES AND PRACTICES IN THE NAME OF OPEN SCIENCE

Editors should implement editorial practices that reinforce adherence to the *modus operandi* of OS in both knowledge production and scientific reporting. This includes promoting open access, shared infrastructure, open data and code, engagement between science and society, and scientific integrity.

## MANIFESTO BY BRAZILIAN JOURNALS IN SUPPORT OF INTEGRATING OPEN SCIENCE PRINCIPLES INTO THE EDITORIAL PROCESS

We, editors of Brazilian scientific journals in the field of aging, encourage researchers in geriatrics, gerontology, and related disciplines to adopt OS principles and practices throughout the entire research cycle, from study conception to dissemination of results. Accordingly, we advocate for:

- The formulation of research questions that address the social needs of older adults and their communities, including the right to age with dignity, while acknowledging the heterogeneity of aging;
- The pursuit of the highest standards of scientific rigor and adherence to best research practices during study conception, through study preregistration, as well as during its development;
- The sharing of infrastructure, data, and open source code with the scientific community;
- The use of national and international shared infrastructures for data collection and analysis, when appropriate;
- The adoption of open licensing;
- The use and reuse of shared datasets;
- The availability of data for verification at the time of manuscript submission, with clear indication of where the data are stored;
- The adoption of clear, transparent, and open editorial policies grounded in ethics and responsibility;
- The promotion of ethical and transparent co-creation practices and citizen science initiatives.

We commit ourselves to this manifesto.

## References

[1] UNESCO Open science [Internet].

[2] Packer A, Santos S (2019). Ciência aberta e o novo modus operandi de comunicar pesquisa – Parte I [Internet].

[3] DORA San Francisco Declaration on Research Assessment [Internet].

[4] Nações Unidas Brasil Declaração Universal dos Direitos Humanos [Internet].

