# Supplementary material for: Manifesto by the editors of Brazilian scientific journals in the field of human aging in support of open access to research and publications
Source: Dement Neuropsychol. 2026 Sep 7;20:e2026E001. doi: 10.1590/1980-5764-DN-2026-E001 (PMC13552992; doi:10.1590/1980-5764-DN-2026-E001)
Supplement: Translation

# Translation: Manifesto dos editores de periódicos científicos brasileiros da área do envelhecimento humano em defesa do acesso aberto a pesquisas e publicações

**DOI:** 10.1590/1980-5764-DN-2026-E001-PT

**Authors:** Patrick Alexander Wachholz, Adriano Pasqualotti, Alfredo Cataldo, Anelise Crippa, Beltrina Côrte, Carla Helena Augustin Schwanke, Einstein Francisco de Camargos, Graciela de Brum Palmeiras, Johannes Doll, Kenio Costa de Lima, Lilian Cristine Hubner, Maria Elisa Gonzalez Manso, Régis Gemerasca Mestriner, Renato Gorga Bandeira de Melo, Ricardo Nitrini, Roberto Alves Lourenço, Sonia Maria Dozzi Brucki, Vanessa Sgnaolin, Renata Eloah de Lucena Ferretti-Rebustini

**Affiliations:** 1 Revista Brasileira de Ciências do Envelhecimento Humano, Passo Fundo RS, Brasil.; 2 Pan-American Journal of Aging Research, Porto Alegre RS, Brasil.; 3 Kairós Gerontologia, São Paulo SP, Brasil.; 4 Geriatrics, Gerontology and Aging, Rio de Janeiro RJ, Brasil.; 5 Estudos Interdisciplinares Sobre o Envelhecimento, Porto Alegre RS, Brasil.; 6 Revista Brasileira de Geriatria e Gerontologia, Rio de Janeiro RJ, Brasil.; 7 Dementia & Neuropsychologia, São Paulo SP, Brasil.

O mundo acadêmico tem se dedicado a compreender e incorporar o *modus operandi* da Ciência Aberta (CA) às práticas de produção e disseminação do conhecimento científico. A Organização das Nações Unidas para a Educação, a Ciência e a Cultura (UNESCO) apresenta a CA como um movimento que busca tornar o conhecimento científico disponível, acessível e reutilizável, com base em eixos temáticos que incluem o acesso aberto, a disponibilização de dados e de códigos, a infraestrutura compartilhável, o engajamento com a sociedade e a integridade científica.^1^

O rápido avanço da ciência exige que as práticas de CA sejam adotadas e consolidadas ao longo de todo o ciclo da pesquisa científica. Por isso, é importante que os periódicos científicos se comprometam com a adoção de boas práticas de CA. Este documento representa um manifesto dos editores de periódicos nacionais dedicados à publicação de investigações e artigos científicos sobre o envelhecimento, em nome da adoção e da implementação dos princípios de CA em seus processos editoriais.

## UM CHAMADO À MUDANÇA DE PARADIGMA

O envelhecimento acelerado da população mundial apresenta diversos desafios a todos os setores da sociedade, incluindo os sistemas de saúde e as instituições de ensino e pesquisa. Responder aos desafios inerentes ao processo de envelhecimento no Brasil e no mundo requer a reorganização de processos e práticas de investigação científica para atender às demandas específicas dessa população. A CA apresenta-se como necessária, diante desse cenário pois, por meio de seus princípios e práticas, apoia a produção de conhecimento científico qualificado baseado em evidências, acessível, compartilhável, verificável, ético, responsável e socialmente útil.

## A CIÊNCIA ABERTA NAS PESQUISAS EM ENVELHECIMENTO HUMANO

O envelhecimento, como fenômeno heterogêneo e multidimensional, impõe a necessidade de cooperação transdisciplinar para produção e disseminação do conhecimento científico. Pesquisas desenvolvidas sob ópticas que não consideram a pluralidade de olhares limitam a compreensão do todo. Nesse sentido, os princípios da CA podem fomentar uma cultura de cooperação e compartilhamento, promovendo a produção de conhecimento específico na fronteira do saber científico, por meio da integração de pesquisadores e diversos setores da sociedade.

A adoção do *modus operandi* da CA visa garantir o acesso gratuito e irrestrito aos resultados de pesquisas científicas, tanto para pesquisadores quanto para toda a sociedade.^2^ Ela garante transparência quanto aos métodos de investigação empregados e ao conteúdo dos dados coletados. Além disso, fortalece a reprodutibilidade das investigações e o emprego de métodos de pesquisa mais avançados, por meio do compartilhamento de artefatos de pesquisa abertos e favorece a avaliação responsável em pesquisa. Por fim, estimula a inclusão de diversos olhares da sociedade, dando voz às pessoas idosas e às comunidades às quais pertencem, e fortalece a confiança da sociedade na ciência.

## O PAPEL DOS PERIÓDICOS CIENTÍFICOS

O papel dos periódicos científicos é estratégico, pois são considerados os principais veículos de disseminação do conhecimento acadêmico, atuando como canais oficiais que validam a qualidade, a originalidade e a relevância de pesquisas antes de sua publicação. Por meio do processo editorial, periódicos recebem, avaliam e disseminam resultados de pesquisas científicas com metodologia rigorosa, apreciada pelos especialistas da comunidade científica. Editores científicos zelam pela disseminação de conhecimento científico qualificado e são, portanto, agentes de mudança.

Ao adotar práticas editoriais que integram os princípios da CA, os periódicos científicos fortalecem a cultura da popularização da ciência, bem como o acesso gratuito e irrestrito a resultados, dados e códigos compartilháveis e verificáveis. Ao adotar práticas de avaliação responsável em pesquisa, os periódicos fortalecem também os princípios da Declaração de São Francisco sobre Avaliação da Pesquisa (DORA).^3^ O resultado esperado é a ampliação do impacto da produção científica e a democratização do saber, contribuindo para que novas descobertas sobre o fenômeno do envelhecimento cheguem a toda a sociedade.

## COMPROMISSO SOCIAL

O acesso à informação é um direito universal previsto na Declaração Universal dos Direitos Humanos.^4^ Essa prerrogativa deve estender-se aos resultados de pesquisas científicas, garantindo que descobertas relevantes sejam compartilhadas com a sociedade para promover o desenvolvimento coletivo. No que tange às investigações sobre o envelhecimento humano, tais preceitos assumem um caráter fundamental, pois são indissociáveis dos princípios de equidade e justiça social. Nesse cenário, a CA contribui decisivamente para mitigar as assimetrias na produção e no acesso ao saber científico, sobretudo em países como o Brasil. Além de viabilizar o ingresso de países menos favorecidos nas infraestruturas globais de pesquisa, a CA consolida a democratização do saber como um imperativo ético do pesquisador. Cabe, portanto, aos periódicos científicos fomentar e salvaguardar tais práticas.

## EM NOME DE UMA NOVA POLÍTICA EDITORIAL

Editores de periódicos científicos são encorajados a adotar os princípios de CA em seus processos e práticas editoriais. Eles devem, inclusive, liderar o movimento de integração da CA no processo editorial, em nome de uma nova cultura editorial, mais equitativa, mais transparente e mais responsável. Se entendemos que a CA catalisa a inovação, editores de periódicos devem ser agentes de mudança. Ao pensarmos em pesquisa sobre envelhecimento que aborde cuidado transdisciplinar, sustentável, digno e de qualidade, esta deve ser desenvolvida e divulgada de portas abertas para o avanço da sociedade.

## ESTÍMULO A PROCESSOS E PRÁTICAS EDITORIAIS EM NOME DA CIÊNCIA ABERTA

Editores científicos devem adotar práticas editoriais que reforcem a necessidade de adesão ao *modus operandi* da CA na produção do conhecimento e na apresentação do relato científico, por meio do estímulo ao acesso aberto, à infraestrutura compartilhada, aos dados e aos códigos abertos, à ciência e sociedade e à integridade científica.

## MANIFESTO DOS PERIÓDICOS BRASILEIROS EM FAVOR DA INTEGRAÇÃO DOS PRINCÍPIOS DA CIÊNCIA ABERTA NO PROCESSO EDITORIAL

Nós, editores de periódicos científicos brasileiros na área do envelhecimento, estimulamos os pesquisadores dos campos de geriatria, gerontologia e áreas afins a adotarem princípios e práticas de CA em todo o ciclo de produção do conhecimento científico, desde a concepção do estudo até a disseminação dos resultados. Por todas as razões acima, estimulamos:

- A formulação de perguntas de pesquisa que respondam às demandas sociais de pessoas idosas e comunidades que as cercam, assim como em relação ao direito de envelhecer com dignidade, e contemplem a heterogeneidade do envelhecimento;
- A busca pelo máximo rigor científico e o zelo pelas boas práticas científicas durante a concepção da pesquisa, por meio de pré-registro do projeto, assim como durante seu desenvolvimento;
- O compartilhamento de infraestrutura, de dados e de códigos abertos com outros pesquisadores;
- A utilização de infraestrutura compartilhável, nacional e internacional, se necessária, para coleta e análise de dados;
- O uso de licenças abertas;
- O uso e reuso de dados compartilhados;
- A disponibilização dos dados para verificação, por ocasião da submissão do manuscrito, indicando o local de compartilhamento;
- A adoção de políticas editoriais claras, transparentes e abertas, com ética e responsabilidade;
- O estímulo a práticas de cocriação e ciência cidadã que sejam éticas e transparentes.

Com este manifesto, nós nos comprometemos.

# Translation: Manifiesto de los editores de revistas científicas brasileñas especializadas en el envejecimiento humano en apoyo del acceso abierto a la investigación y las publicaciones

**DOI:** 10.1590/1980-5764-DN-2026-E001-ES

**Authors:** Patrick Alexander Wachholz, Adriano Pasqualotti, Alfredo Cataldo, Anelise Crippa, Beltrina Côrte, Carla Helena Augustin Schwanke, Einstein Francisco de Camargos, Graciela de Brum Palmeiras, Johannes Doll, Kenio Costa de Lima, Lilian Cristine Hubner, Maria Elisa Gonzalez Manso, Régis Gemerasca Mestriner, Renato Gorga Bandeira de Melo, Ricardo Nitrini, Roberto Alves Lourenço, Sonia Maria Dozzi Brucki, Vanessa Sgnaolin, Renata Eloah de Lucena Ferretti-Rebustini

**Affiliations:** 1 Revista Brasileira de Ciências do Envelhecimento Humano, Passo Fundo RS, Brasil.; 2 Pan-American Journal of Aging Research, Porto Alegre RS, Brasil.; 3 Kairós Gerontologia, São Paulo SP, Brasil.; 4 Geriatrics, Gerontology and Aging, Rio de Janeiro RJ, Brasil.; 5 Estudos Interdisciplinares Sobre o Envelhecimento, Porto Alegre RS, Brasil.; 6 Revista Brasileira de Geriatria e Gerontologia, Rio de Janeiro RJ, Brasil.; 7 Dementia & Neuropsychologia, São Paulo SP, Brasil.

El mundo académico se ha dedicado a comprender e incorporar el *modus operandi* de la Ciencia Abierta (CA) en las prácticas de producción y difusión del conocimiento científico. La Organización de las Naciones Unidas para la Educación, la Ciencia y la Cultura (UNESCO) presenta la CA como un movimiento que busca que el conocimiento científico esté disponible, sea accesible y reutilizable, basándose en ejes temáticos que incluyen el acceso abierto, la disponibilidad de datos y códigos, la infraestructura compartida, el compromiso con la sociedad y la integridad científica.^1^

El rápido avance de la ciencia exige que las prácticas de CA se adopten y consoliden a lo largo de todo el ciclo de la investigación científica. Por ello, es importante que las revistas científicas se comprometan con la adopción de buenas prácticas de CA. El presente documento constituye un manifiesto de los editores de revistas nacionales dedicadas a la publicación de investigaciones y artículos científicos sobre el envejecimiento, en favor de la adopción e implementación de los principios de CA en sus procesos editoriales.

## UN LLAMAMIENTO AL CAMBIO DE PARADIGMA

El envejecimiento acelerado de la población mundial plantea diversos retos a todos los sectores de la sociedad, incluidos los sistemas de salud y las instituciones de enseñanza e investigación. Responder a los retos inherentes al proceso de envejecimiento en Brasil y en el mundo requiere la reorganización de los procesos y de las prácticas de investigación científica para atender a las demandas específicas de esta población. La CA se presenta como necesaria en este escenario, ya que, a través de sus principios y prácticas, apoya la producción de conocimiento científico cualificado, basado en la evidencia, accesible, compartible, verificable, ético, responsable y socialmente útil.

## LA CIENCIA ABIERTA EN LA INVESTIGACIÓN SOBRE EL ENVEJECIMIENTO HUMANO

El envejecimiento, como fenómeno heterogéneo y multidimensional, impone la necesidad de una cooperación transdisciplinaria para la producción y difusión del conocimiento científico. Las investigaciones desarrolladas desde perspectivas que no tienen en cuenta la pluralidad de puntos de vista limitan la comprensión del fenómeno en su conjunto. En este sentido, los principios de la ciencia abierta pueden fomentar una cultura de cooperación e intercambio, promoviendo la producción de conocimiento específico en la vanguardia del saber científico, mediante la integración de investigadores y de diversos sectores de la sociedad.

La adopción del *modus operandi* de la CA tiene por objeto garantizar el acceso gratuito e irrestricto a los resultados de la investigación científica, tanto para los investigadores como para toda la sociedad.^2^ Asimismo, garantiza la transparencia en cuanto a los métodos de investigación empleados y al contenido de los datos recopilados. Además, refuerza la reproducibilidad de las investigaciones y el uso de métodos de investigación más avanzados mediante el intercambio de artefactos de investigación abiertos, y favorece la evaluación responsable en la investigación. Por último, fomenta la inclusión de diversas perspectivas de la sociedad, dando voz a las personas mayores y a las comunidades a las que pertenecen, y refuerza la confianza de la sociedad en la ciencia.

## EL PAPEL DE LAS REVISTAS CIENTÍFICAS

El papel de las revistas científicas es estratégico, ya que se consideran los principales vehículos de difusión del conocimiento académico, actuando como canales oficiales que validan la calidad, la originalidad y la relevancia de las investigaciones antes de su publicación. A través del proceso editorial, las revistas reciben, evalúan y difunden los resultados de investigaciones científicas con una metodología rigurosa, valorada por especialistas de la comunidad científica. Los editores científicos velan por la difusión de conocimiento científico cualificado y son, por lo tanto, agentes de cambio.

Al adoptar prácticas editoriales que integran los principios de la CA, las revistas científicas fortalecen la cultura de la divulgación de la ciencia, así como el acceso gratuito e irrestricto a resultados, datos y códigos compartibles y verificables. Asimismo, al adoptar prácticas de evaluación responsable en la investigación, las revistas refuerzan los principios de la Declaración de San Francisco sobre la Evaluación de la Investigación (DORA).^3^ El resultado esperado es la ampliación del impacto de la producción científica y la democratización del conocimiento, contribuyendo a que los nuevos descubrimientos sobre el fenómeno del envejecimiento lleguen a toda la sociedad.

## COMPROMISO SOCIAL

El acceso a la información es un derecho universal previsto en la Declaración Universal de los Derechos Humanos.^4^ Esta prerrogativa debe extenderse a los resultados de las investigaciones científicas, garantizando que los descubrimientos relevantes se compartan con la sociedad para promover el desarrollo colectivo. En lo que respecta a las investigaciones sobre el envejecimiento humano, tales preceptos adquieren un carácter fundamental, ya que son indisociables de los principios de equidad y justicia social. En este contexto, la CA contribuye de manera decisiva a mitigar las asimetrías en la producción y el acceso al conocimiento científico, especialmente en países como Brasil. Además de facilitar la incorporación de los países menos favorecidos a las infraestructuras globales de investigación, la CA consolida la democratización del conocimiento como un imperativo ético del investigador. Corresponde, por tanto, a las revistas científicas fomentar y salvaguardar dichas prácticas.

## EN NOMBRE DE UNA NUEVA POLÍTICA EDITORIAL

Se anima a los editores de revistas científicas a adoptar los principios de la CA en sus procesos y prácticas editoriales. Deben, incluso, liderar el movimiento de integración de la CA en el proceso editorial, en nombre de una nueva cultura editorial, más equitativa, transparente y responsable. Si se entiende que la CA cataliza la innovación, los editores de revistas deben ser agentes de cambio. Al considerar la investigación sobre el envejecimiento que aborde el cuidado transdisciplinario, sostenible, digno y de calidad, esta debe desarrollarse y difundirse con las puertas abiertas al avance de la sociedad.

## FOMENTO DE PROCESOS Y PRÁCTICAS EDITORIALES EN NOMBRE DE LA CIENCIA ABIERTA

Los editores científicos deben adoptar prácticas editoriales que refuercen la necesidad de adherirse al *modus operandi* de la CA en la producción de conocimiento y en la presentación del informe científico, mediante el fomento del acceso abierto, la infraestructura compartida, los datos y códigos abiertos, la ciencia y la sociedad, y la integridad científica.

## MANIFIESTO DE LAS REVISTAS BRASILEÑAS A FAVOR DE LA INTEGRACIÓN DE LOS PRINCIPIOS DE LA CIENCIA ABIERTA EN EL PROCESO EDITORIAL

Los editores de revistas científicas brasileñas en el ámbito del envejecimiento animan a los investigadores de los campos de la geriatría, la gerontología y áreas afines a adoptar los principios y prácticas de la CA en todo el ciclo de producción del conocimiento y del informe científico, desde la concepción del estudio hasta la difusión de los resultados. Por todas las razones anteriores, se promueve:

- La formulación de preguntas de investigación que respondan a las demandas sociales de las personas mayores y de las comunidades que las rodean, así como al derecho a envejecer con dignidad, teniendo en cuenta la heterogeneidad del envejecimiento;
- La búsqueda del máximo rigor científico y la observancia de las buenas prácticas científicas durante la concepción de la investigación, mediante el prerregistro del proyecto, así como durante su desarrollo;
- El intercambio de infraestructura, datos y códigos abiertos con otros investigadores;
- El uso de infraestructura compartida, nacional e internacional, cuando sea necesario, para la recopilación y el análisis de datos;
- El uso de licencias abiertas;
- El uso y la reutilización de datos compartidos;
- La puesta a disposición de los datos para su verificación, en el momento de la presentación del manuscrito, indicando el lugar de intercambio;
- La adopción de políticas editoriales claras, transparentes y abiertas, con ética y responsabilidad;
- El fomento de prácticas de cocreación y ciencia ciudadana que sean éticas y transparentes.

Con este manifiesto, se asume el compromiso.
